# New Insights into D-Aspartate Signaling in Testicular Activity †

**DOI:** 10.3390/cells13161400

**Published:** 2024-08-22

**Authors:** Sara Falvo, Alessandra Santillo, Maria Maddalena Di Fiore, Massimo Venditti, Giulia Grillo, Debora Latino, Isabella Baccari, Giuseppe Petito, Gabriella Chieffi Baccari

**Affiliations:** 1 Department of Environmental, Biological and Pharmaceutical Sciences and Technologies, University of Campania ‘Luigi Vanvitelli’, 81100 Caserta, Italy; sara.falvo@unicampania.it (S.F.); alessandra.santillo@unicampania.it (A.S.); giulia.grillo@unicampania.it (G.G.); debora.latino@unicampania.it (D.L.); giuseppe.petito@unicampania.it (G.P.); gabriella.chieffi@unicampania.it (G.C.B.); 2 Department of Experimental Medicine, University of Campania ‘Luigi Vanvitelli’, 80138 Napoli, Italy; massimo.venditti@unicampania.it (M.V.); isabellabaccari@gmail.com (I.B.)

**Keywords:** D-aspartate, reproduction, testis, spermatogenesis, steroidogenesis, ERK1/2, AKT, mitochondria, MAMs, D-amino acids

## Abstract

D-aspartate (D-Asp) is an amino acid found in high concentrations in the testis and pituitary gland. Increasing evidence suggests that D-Asp promotes spermatogenesis by activating testosterone production in the Leydig cells via LH release from the pituitary gland. In vitro studies indicate that D-Asp may also influence steroidogenesis and spermatogenesis through autocrine and paracrine signals. D-Asp enhances StAR and steroidogenic enzyme expressions, facilitating testicular cell proliferation via the GluR/ERK1/2 pathway. Moreover, it supports spermatogenesis by enhancing the mitochondrial function in spermatocytes, aiding in the metabolic shift during meiosis. Enhanced mitochondrial function, along with improved MAM stability and reduced ER stress, has been observed in Leydig and Sertoli cells treated with D-Asp, indicating potential benefits in steroidogenesis and spermatogenesis efficiency. Conversely, D-Asp exerts a notable anti-apoptotic effect in the testis via the AMPAR/AKT pathway, potentially mediated by antioxidant enzyme modulation to mitigate testicular oxidative stress. This review lays the groundwork for future investigations into the molecules promoting spermatogenesis by stimulating endogenous testosterone biosynthesis, with D-amino acids emerging as promising candidates.

## 1. Introduction

Spermatogenesis is an extremely complex process that is highly regulated, not only by the endocrine system but also by paracrine factors in the surrounding microenvironment. Understanding the mechanisms that orchestrate male gamete differentiation is essential for better intervention in male infertility, a major cause of reduced reproduction worldwide.

Over the past 20 years, improvements in analytical methods have revealed that free D-amino acids, D-aspartate (D-Asp), and D-serine (D-Ser) are commonly present in vertebrate testes [1,2,3,4,5,6]. D-Asp and D-Ser bind to glutamate receptors (GluRs) and function as coagonists and agonists, respectively [7,8,9,10,11]. D-Asp and D-Ser are the only D-amino acids known to originate from racemization of the corresponding L-amino acids by tissue-intrinsic racemases [12]. The activity of aspartate racemase, a pyridoxal 5′-phosphatase-dependent enzyme that converts L-Asp to D-Asp, has been detected in the rat pituitary gland [13,14] and in the testis [14,15]. Studies carried out in seasonal reproductive vertebrates demonstrated that testicular racemase activity peaks in the reproductive period, when testosterone levels are at their highest, during spermatogenesis and reproduction. Thus, at least a some of the D-Asp formed in the testis during the reproductive phase can contribute to the formation of more testosterone [16]. Conversely, L-Asp did not affect testicular activity. 

The involvement of D-Asp in the reproductive process was discovered approximately 30 years ago. The first investigations were focused on the major vertebrate classes, from amphibian to human, and demonstrated that D-Asp acts at all levels of the hypothalamic–pituitary–testis axis [1,4]. D-Asp functions as an excitatory molecule by stimulating the synthesis of GnRH in the hypothalamus, LH in the adenohypophysis, and testosterone in the Leydig cells (LCs) [1,17,18,19,20]. Thus, D-Asp can enhance spermatogenesis by indirectly promoting testosterone biosynthesis. A correlation between endogenous levels of D-Asp and testosterone concentrations has been observed in the rat testis across the lifespan [21,22]. Initially, both D-Asp and testosterone levels are low at birth and gradually increase, reaching peak levels at sexual maturity. 

A recent study using Ddo knock-in mice has further proved a correlation between D-Asp levels and testosterone concentrations [23]. Ddo knock-in mice are characterized by elevated levels of D-aspartate oxidase (DDO), the enzyme responsible for the oxidative deamination of D-Asp [24]. In Ddo knock-in mice, a marked reduction in testicular D-Asp levels was observed, which was accompanied by a significant decrease in serum testosterone levels [23]. This recognition of the important role of D-Asp in testosterone biosynthesis paved the way for the development of D-Asp-based pharmaceutical preparations, which have been successfully used to treat human male infertility [18].

A comprehensive biochemical analysis conducted by D’Aniello et al. [25] examined various compartments of the rat testis. Their study revealed that D-Asp is most concentrated in testicular venous plasma, followed by the testicular network, the testicular parenchymal cells, the luminal fluid of the seminiferous tubules, and the interstitial extracellular fluid. Other studies have also identified D-Asp in both germ cells and somatic cells in rodents [22], and human spermatozoa [26], indicating its potential role in regulating cell function through autocrine and/or paracrine signals. More recent in vitro studies carried out on testicular germ and somatic cell lines have facilitated an understanding of the molecular pathways activated by D-Asp in both steroidogenesis and spermatogenesis [27,28,29,30]. 

A parallel line of research, which would be important and interesting to develop in the future and may contribute to the understanding of testicular function in the presence of the different free amino acid derivatives, concerns D-Ser. D-Ser levels in the rat testis are initially high at birth, decrease significantly by the end of the first week, and then stabilize at minimal levels throughout subsequent development [31]. Recent research indicates that D-Ser, together with retinoic acid, stimulates spermatogonial proliferation via the NMDAR-dependent activation of ERK and Akt signaling pathways [32]. Additionally, a study by Noghani et al. [33] demonstrated that D-Ser enhances the expression of premeiotic (Plzf), meiotic (Sycp3), and postmitotic (Tnp1) genes and proteins in decellularized testicular matrix (DTM) hydrogel scaffolds. Nevertheless, further research is necessary to fully elucidate the role of D-Ser in testicular function. 

This review aims to summarize the latest findings regarding D-Asp as a regulator of male reproductive processes, emphasizing its impact on testicular germ and somatic cells at the molecular level. We believe this review will establish a solid foundation for future research on D-amino acids in reproductive biology, contributing to the theoretical framework and clinical approaches for male infertility disorders.

## 2. D-Asp Regulates the Functions of Leydig Cells via the cAMP/ERK1/2 Signaling Pathway

**Figure 1:**
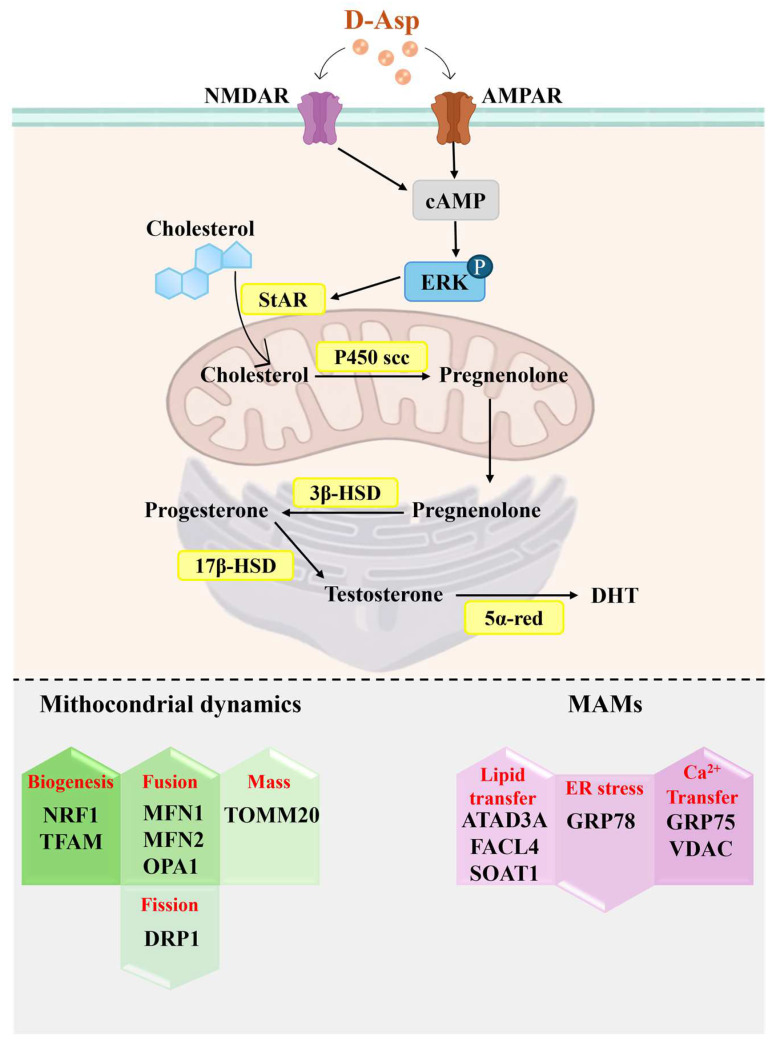
**Effects of D-Asp on Leydig cell function.** In Leydig cells, D-Asp promotes the phosphorylation of ERK via NMDAR/AMPAR/cAMP. ERK activates gene transcription/protein expression of StAR and steroidogenic enzymes (P450scc, 3β-HSD, 17β-HSD, 5α-red), resulting in the biosynthesis of androgen hormones (testosterone; dihydrotestosterone, DHT). D-Asp also enhances the expression of proteins involved in mitochondrial biogenesis (NRF1, TFAM), fusion (MFN1, MFN2, OPA1), and mass (TOMM20), and reduces the expression of DRP1, a marker of mitochondrial fission. Finally, the amino acid induces an increase in the expression of proteins involved in MAM structure stabilization (ATAD3A, FACL4, SOAT1, VDAC, GRP75), and a reduction in the expression of GRP78, which is indicative of a decrease in ER stress.

Leydig cells are recognized as the primary source of testosterone in the testis. Testosterone, a crucial androgen, plays a pivotal role in promoting spermatogenesis [34]. The incidence of male infertility cases attributable to reduced testosterone levels is increasing. However, exogenous testosterone typically leads to the suppression of LH secretion from the pituitary gland, thereby reducing testosterone production by LCs [35,36]. Consequently, enhancing endogenous testosterone levels is crucial in cases of male infertility caused by testosterone deficiency. In this context, a growing body of in vivo research in rodents has demonstrated that D-Asp, naturally present in the testis and pituitary, stimulates testosterone biosynthesis in LCs by inducing LH release from the pituitary [14,18,20,37,38,39]. Supporting this, an upregulation in the gene/protein expression of the steroidogenic acute regulatory protein (StAR), a mitochondrial cholesterol transport protein, and steroidogenic enzymes (P450 cholesterol side chain cleavage, P450scc; 3β-Hydroxysteroid dehydrogenase, 3β-HSD; 17β-Hydroxysteroid dehydrogenase, 17β-HSD; 5α-reductase, 5α-red) has been observed in the rat testis following D-Asp treatment (oral administration of 20 mM D-Asp for 15 days) [17,37,38] (Figure 1). In Ddo knock-in mice, which exhibit low testicular concentrations of D-Asp, both serum and testicular testosterone levels, as well as the expression of 17β-HSD (the enzyme catalyzing the conversion of androstenedione to testosterone), were lower compared to wild-type mice. This indicated that D-Asp depletion may alter steroidogenesis [23].

However, in vitro studies of LCs have shown that D-Asp can also promote steroidogenesis through autocrine and/or paracrine regulation [37,40,41]. In LCs, D-Asp alone or with human chorionic gonadotropin can directly activate testosterone biosynthesis by upregulating the expression of StAR mRNA/protein, P450scc mRNA, which catalyzes the conversion of cholesterol to pregnenolone, and 3β-HSD mRNA, which catalyzes the conversion of progesterone from pregnenolone (Figure 1). In addition, Topo et al. [14] reported that rat LCs incubated with 10 mM D-Asp showed a significant increase in cAMP levels, which in turn affected the phosphorylation of ERK1/2, resulting in increased androgen secretion (Figure 1). ERK is a mitogen-activated protein kinase that belongs to a family of serine/threonine kinases occupying a focal point in signal transduction, participating in the activation of gene transcription via translocation into the nucleus [42]. The ERK1/2 belongs to the cascade of RAS/RAF/MEK/ERK reactions within the MAPK signaling pathway.

Notably, it has been widely shown that the ERK1/2 pathway regulates androgen secretion in LCs by influencing the transcription of related molecules and their phosphorylation levels [36,43]. In this context, it has been demonstrated that certain hormones and factors, such as LH, epidermal growth factor (EGF), and the membrane-bound protein A5, can stimulate testosterone production by activating the ERK1/2 pathway [44]. Moreover, it has been suggested that the inhibition of testosterone secretion in LCs induced by nano-TiO2 may arise from dysfunction in the ERK1/2/PKA/PKC signaling pathway, leading to a downregulation in StAR, P450scc, 3β-HSD, SR-BI, PKA, PKC, and p-ERK1/2 expression [45]. Our in vitro study conducted on Leydig TM3 cells demonstrated that the addition of D-Asp to the incubation medium activated the ERK1/2 pathway [46]. Based on the above findings, it can be inferred that D-Asp activates testosterone production in LCs by upregulating steroidogenesis enzymes via the ERK1/2 pathway. Since rat LCs express GluR 2/3 (AMPAR subunit) and NMDAR [47,48], it is hypothesized that D-Asp activates the ERK pathway via GluRs (Figure 1).

Finally, mitochondria-associated endoplasmic reticulum membranes (MAMs) mediate communication between the endoplasmic reticulum (ER) and the mitochondria, playing a fundamental role in steroidogenesis. Notably, a recent study showed that D-Asp promotes testosterone biosynthesis by modulating MAMs and regulating lipid trafficking, calcium signaling, ER stress, and mitochondrial dynamics [39]. Briefly, in the testes of rats orally treated with D-Asp, increases in the expression levels of proteins involved in lipid transfer (ATPase family AAA domain-containing 3A, ATAD3A; long-chain-fatty-acid-CoA ligase, FACL4; and sterol O-acyltransferase 1, SOAT1) and proteins involved in calcium signaling (voltage-dependent anion channels, VDAC, and dynamin-related protein 1, GRP75) were detected (Figure 1). In addition, a decrease in the protein expression levels of glucose-regulated protein 78 (GRP78), an enzyme that counteracts ER stress, was detected. D-Asp also affects the mitochondrial compartment by inducing a significant increase in the expression of proteins involved in fusion (mitofusin 1, MFN1, mitofusin 2, MFN2, and optic atrophy type 1, OPA1) and biogenesis (nuclear respiratory factor 1, NRF1, and mitochondrial transcription factor A, TFAM), as well as in mitochondrial mass (translocase of the outer mitochondrial membrane complex subunit 20, TOMM20) (Figure 1). Immunohistochemical analysis confirmed the localization of the abovementioned markers in LCs by showing an increase in intensity in D-Asp-treated rat testes [39]. Furthermore, reduced expression of DRP1 (Dynamin-related protein 1), a key regulator of mitochondrial fission, was described in the LCs of D-Asp treated rats (Figure 1). 

Taken together, these results strongly support the role of D-Asp as a signaling molecule in steroidogenesis activation.

## 3. D-Asp Activates Spermatogenesis via the GluR-ERK1/2 Signaling Pathway

**Figure 2:**
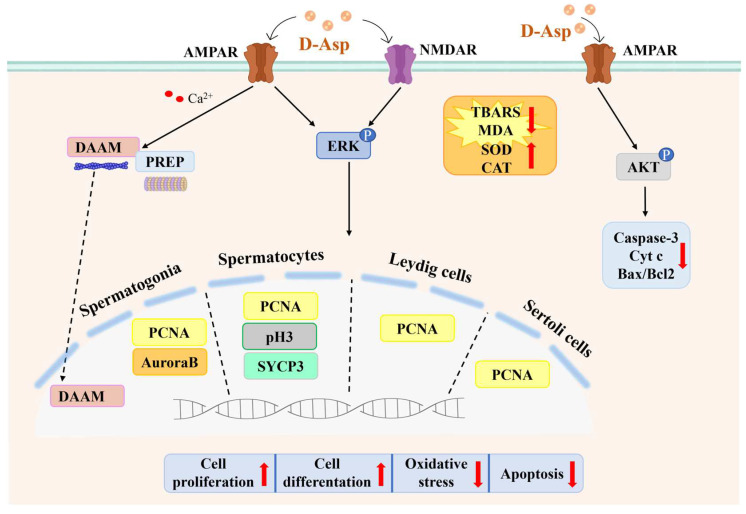
**D-Asp modulates spermatogenesis through the GluR/ERK1/2/AKT pathways.** D-Asp activates the ERK/AKT pathway via GluRs (NMDAR and AMPAR) in testicular germ cells (spermatogonia and spermatocytes) and somatic cells (Leydig and Sertoli cells). ERK activates the expression of proteins involved in mitotic and meiotic processes (PCNA, AuroraB, pH3, SYCP3). D-Asp, via the AMPAR-dependent molecular pathway, elicits an increase in PREP protein by mediating calcium signaling; in spermatogonia the amino acid upregulates the expression of DAAM protein and mediates its transfer into the nucleus. Finally, D-Asp increases the levels of antioxidant enzymes (SOD, CAT), and induces a decrease in TBARS and MDA, resulting in a reduction in oxidative status; by promoting AKT phosphorylation, the amino acid inhibits the apoptotic process, as evidenced by decreased expression levels of Cyt c and caspase-3 and a decreased Bax/Bcl2 ratio.

Several lines of evidence indicate the ability of D-Asp to stimulate the same excitatory receptors as L-glutamate, namely, GluRs [8,9,10,11]. Various subtypes of ionotropic GluRs (iGluRs), including NMDA, AMPA, and kainate receptors, as well as metabotropic GluRs (mGluRs), are expressed in rat and mouse testes [47,48,49,50,51,52]. The NMDA receptor forms as a heteromeric tetramer consisting of the obligatory NR1 subunit and modulatory NR2 subunits (NR2A, NR2B, NR2C, and NR2D), each encoded by distinct genes [53]. In the rat testis, NR1, NR2A-C, and NR2D subunits are expressed [51], while in mice, NR1 and NR2B subunits are expressed [54]. Treatment of rats with D-Asp (oral administration of 20 mM D-Asp for 15 days) significantly enhances the testicular expression of NR1 and NR2A mRNAs [55]. Additional in vivo experiments have demonstrated that D-Asp induces elevated expression of the GluA2/3 subunit of AMPAR in rat LCs and germinal epithelium [56]. In agreement, in vitro studies on the mouse cell line GC-1, a cell line derived from B-type spermatogonia [57], and on mouse GC-2, an intermediate stage between preleptotene spermatocytes and round spermatids [29], showed that D-Asp acts on both the GluA1 and GluA2/3 subunits of the AMPAR (Figure 2).

The activation of NMDARs and AMPARs induces the phosphorylation of ERK. Considerable evidence indicates that ERK1/2 is ubiquitously distributed and activated at multiple sites in the male reproductive system and plays an important role in various stages of male reproduction, including the development and regulation of male germ cell proliferation/maturation [36]. ERK1/2 is activated at the G2/M transition and may play a role in meiosis by regulating the genes involved in this process. Indeed, the loss of SHP2, an important regulator of ERK1/2 signaling, suppresses the phosphorylation of ERK and thus the expression of the meiotic genes Sycp3 and Dmc1; conversely, deletion of the L-GILZ gene increases ERK1/2 signaling and accelerates the proliferation of undifferentiated spermatogonia [58].

Administration of D-Asp in vivo leads to ERK1/2 phosphorylation in the rat testis [47,55,56] (Figure 2). Similarly, in vitro studies have demonstrated that D-Asp induces the phosphorylation of ERK1/2 proteins in GC-1 cells [27,57], GC-2 cells [29], TM4 Sertoli cells [30], and, as previously reported, TM3 cells [46]. Consistent findings from in vivo and in vitro experiments show an increased protein expression of proliferation markers following D-Asp treatment (Figure 2). Specifically, D-Asp treatment has been associated with elevated levels of (1) proliferating cell nuclear antigen (PCNA), a nuclear protein expressed during the S phase of the cell cycle, in the rat testis [28,38], GC-1 cells [27], GC-2 cells [29], TM4 cells [30], and TM3 cells [46]; (2) Aurora B, a critical regulator of mitosis essential for genome stability and G2-M transition, in GC-1 cells [57]; and (3) pH3, a histone protein crucial for chromatin condensation during mitosis and meiosis, SYCP3, which encodes the proteins involved in forming the synaptonemal complex, in the rat testis [38] and GC-2 cells [29] (Figure 2). Therefore, the increased expression of proliferative markers in both testicular germ and somatic cells following D-Asp treatment strongly supports the hypothesis that this amino acid plays a crucial role in promoting spermatogenesis via the ERK pathway. Supporting evidence includes the findings from Ddo knock-in mice, which exhibit low testicular D-Asp concentrations and reduced levels of PCNA and SYCP3 compared to wild-type mice, indicating that D-Asp depletion alters spermatogenesis [23]. Lastly, the observed increase in GluR (NMDA-AMPAR) expression following D-Asp treatment in rat testes, as well as in GC-1 cells [27,56,57] and GC-2 cells [29], suggests that this amino acid may influence spermatogenesis via the GluR-ERK pathway (Figure 2). 

D-Asp has been demonstrated to promote spermatogenesis by inducing cytoskeleton remodeling through the modulation of protein expression, including prolyl endopeptidase (PREP) and disheveled-associated activator of morphogenesis 1 (DAAM) [28,56,59] (Figure 2). PREP, a serine protease expressed in the cytoplasm and nucleus of germ cells and Sertoli cells (SCs), participates in microtubule-associated processes, which are crucial for cytoskeletal remodeling during germ cell movement [60]. Studies using PREP-knockdown mice have shown significant alterations in the gonadal structure and spermatogenic function, highlighting the essential role of PREP in normal spermatogenesis [61]. Administration of D-Asp, both chronically and acutely, to adult rats increases PREP protein expression in the testes [56]. Importantly, immunofluorescence analyses have revealed the co-localization of PREP with GluA2/3 in the rat testis [56]. The GluA2 subunit regulates the calcium permeability of AMPARs [56,62], suggesting that PREP may facilitate the AMPAR-dependent pathway activated by D-Asp through calcium signaling (Figure 2). Furthermore, recent research in Ddo knock-in mice has confirmed the role of D-Asp in regulating PREP expression [23]. These mice exhibit elevated testicular PREP protein levels, predominantly localized in the cytoplasmic extensions of SCs, which are crucial for the proper microtubule-mediated movement of the germ cells across the seminiferous epithelium. The increased PREP levels observed in the SCs of Ddo knock-in mice may represent a compensatory mechanism against microtubule network disorganization, a contributing factor to impaired spermatogenesis in these animals [23].

Cell differentiation during spermatogenesis requires proper actin dynamics, which are regulated by several proteins including DAAM1, which is a formin that promotes actin polymerization. Venditti et al. [28] showed that oral administration of D-Asp increases DAAM1 protein levels in the cytoplasm of rat testis germ cells and hypothesized that this effect occurs via AMPAR (Figure 2). Interestingly, after treatment with D-Asp, DAAM1 also localized to the nucleus of spermatogonia, suggesting that D-Asp in these cells promotes the shuttling of DAAM1 to the nuclear compartment (Figure 2). DAAM1 plays a dual role in B-type spermatogonia; in the cytoplasm, it regulates actin remodeling during germ cell differentiation, and in the nucleus, it enables actin polymerization, which is important for DNA replication and ultimately for cell division [28]. Finally, Santillo et al. [23] reported that the protein level of DAAM1 in the testes of Ddo knock-in mice was lower than that in the testes of wild-type mice. Therefore, a decrease in DAAM1 expression triggered by a lack of D-Asp could contribute to the reduced spermatogenesis observed in the testes of Ddo knock-in mice.

Thus, D-Asp participates in the proliferation of the testicular cells (germ cells and somatic cells) and in the differentiation of the spermatogenic cells by activating the GluR/ERK1/2 pathway. Furthermore, D-Asp could contribute to spermatogenesis through the modulation of PREP and DAAM expression, which are proteins involved in cytoskeleton remodeling.

### D-Asp Is Involved in the Metabolic Shift Occurring during Meiosis

During the male germ cell maturation process, the mitochondria remain relatively inactive during the early stages of germ cell development and then become progressively more active as maturation progresses [63,64]. According to this model, the spermatogonia exhibit increased glycolytic activity, while the spermatocytes and the spermatids synthesize ATP, mainly through the oxidative phosphorylation complex (OXPHOS) pathway [65,66,67]. Therefore, the metabolic changes that occur during meiosis require increased mitochondrial content (biogenesis and fission), mitochondrial elongation (fusion), and increased levels of OXPHOS [68,69]. The OXPHOS complexes are responsible for a series of reversible oxidation–reduction reactions involving nicotinamide adenine dinucleotide (NAD+/NADH) or flavin adenine dinucleotide (FAD/FADH2) molecules, which generate a proton gradient across the intermembrane space of the organelles necessary for ATP production.

Studies conducted on GC-2 cells, an intermediate stage between the preleptotene spermatocytes and the round spermatids, have demonstrated that treatment with D-Asp induces a notable increase in mitochondrial biogenesis. This is evidenced by elevated expression levels of PGC-1α (peroxisome proliferator-activated receptor-gamma coactivator-1alpha), NRF1, and TFAM [29]. Additionally, lysates from these cells revealed an increased expression of MFN2 and OPA1, proteins crucial for outer and inner mitochondrial membrane fusion, respectively, and a reduced expression of DRP1, a key regulator of mitochondrial fission [29]. The essential role of mitochondrial fusion in the metabolic transition during spermatogenesis was underscored in a study in which male germ cell lines lacking MFN1 and MFN2 ceased spermatogenesis immediately upon mitofusin depletion [70]. This suggests that optimal levels of mitochondrial fusion are vital for the cellular physiology of meiotic spermatocytes.

Furthermore, the positive impact of D-Asp on mitochondrial function was illustrated by the increased expression of certain OXPHOS complexes (CIV and CV) in D-Asp-treated GC-2 cells [29]. Recent in vivo research in adult rats further supported D-Asp’s active role in enhancing mitochondrial functionality during spermatogenesis [39]. Specifically, oral administration of D-Asp (20 mM D-Asp for 15 days) significantly elevated the protein expression levels involved in fusion (MFN1, MFN2, OPA1), biogenesis (NRF1, TFAM), and mitochondrial mass (TOMM20), while reducing DRP1 expression. Immunohistochemical analysis indicated increased marker positivity predominantly in the spermatocytes, affirming D-Asp’s role in facilitating the metabolic shift during meiosis [39].

Thus, D-Asp enhances spermatogenesis by improving the mitochondrial functionality that is critical for the metabolic changes that occur during meiosis.

## 4. D-Asp Modulates Oxidative Stress in the Testis

**Figure 3:**
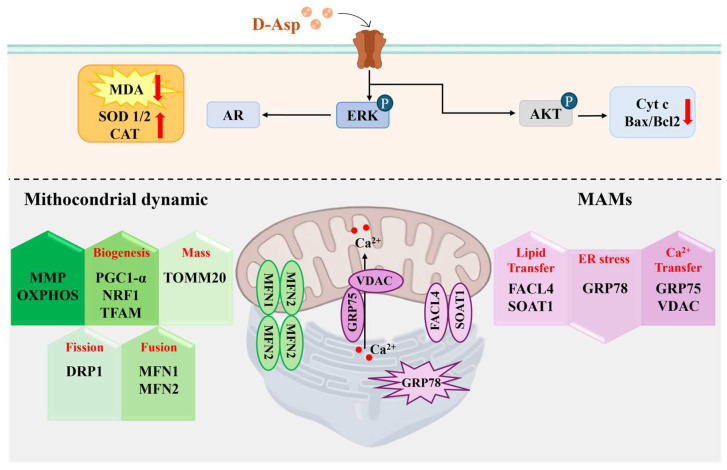
**Effects of D-Asp on Sertoli cell function.** D-Asp influences the activity of TM4 Sertoli cells via the ERK/AKT pathway. The phosphorylation of ERK induces an increase in the expression of AR, and vice versa, the phosphorylation of AKT provokes a reduction in the expression of apoptotic markers (Cyt c and the Bax/Bcl2 ratio). Furthermore, D-Asp enhances the MMP and the expression of OXPHOS and proteins involved in mitochondrial biogenesis (PGC1α, NRF1, and TFAM), mass (TOMM20), fission (DRP1), and fusion (MFN1 and MFN2). D-Asp promotes an increase in the expression of the proteins involved in MAM structure stabilization (GRP75, VDAC, FACL4, SOAT1) and a reduction in GRP78 expression, which is indicative of a decrease in ER stress. Finally, a reduction of oxidative stress, as indicated by decreased MDA levels and increased SOD1/2 and CAT expression levels, was described in D-Asp-treated TM4 cells.

Although reactive oxygen species (ROS) are necessary for the maintenance of spermatogonial stem cells [64,71], the cellular levels of ROS must be tightly regulated to maintain normal cellular function. Indeed, excessive ROS overloads the cellular antioxidant capacity and may result in oxidative damage, leading to apoptosis [72]. Considerable experimental evidence suggests that D-Asp plays a role in reducing oxidative stress in the testicular parenchyma. In vivo administration of D-Asp to adult rats (oral administration of 20 mM D-Asp for 15 days) significantly reduced the testicular oxidative status, as evidenced by a reduction in the level of TBARS, a byproduct of lipid peroxidation, and by stimulating the enzymatic activity/protein expression of the antioxidant enzymes superoxide dismutase (SOD) and catalase (CAT) [39] (Figure 2). In addition, D-Asp can counteract/prevent the oxidative stress induced by ethane dimethane sulfonate [59] and cadmium in rat testes [38] by affecting the SOD and CAT activities and the TBARS levels. A decrease in MDA levels and increased protein expression of CAT, SOD1, and SOD2 were also demonstrated in Sertoli TM4 cells following treatment with D-Asp [30] (Figure 2 and Figure 3).

In agreement with the above, in vitro studies have shown that the addition of D-Asp to the seminal fluid of oligoasthenospermic men reduces oxidative stress, DNA fragmentation, and lipid peroxidation, improving sperm quality [18,73]. In addition, Giacone et al. [74] reported that D-Asp reduced sperm lipid peroxidation in both normospermic and asthenospermic men.

Based on these findings, we confirmed that D-Asp reduces the testicular oxidative status by modulating the protein expression/activity of antioxidant enzymes. Furthermore, some studies suggest that D-Asp may be a promising candidate for alleviating the oxidative stress induced by environmental pollutants in the testis.

## 5. D-Asp Inhibits Apoptosis via the AMPAR/AKT Signaling Pathway

AKT has been reported to mediate cell survival by inactivating several proapoptotic molecules [75,76]. D-Asp stimulates AKT activity in the rat testes [57], GC-1 cells [27], and GC-2 cells [29] via AMPAR [57] (Figure 2). Consistently, oral administration of D-Asp to adult rats (20 mM D-Asp for 15 days) resulted in a decrease in the testicular expression levels of cytochrome c (Cyt c) and caspase-3, and reduced the number of apoptotic cells [38,77] (Figure 2). Furthermore, in D-Asp-treated TM4 cells, an increase in p-AKT and a decrease in Cyt c and the Bax/Bcl2 protein ratio, as well as a reduction in the percentage of DAPI-positive nuclei (see Section 6, Figure 3), are indicative of an inhibition of the apoptosis process in these cells [30]. Finally, in support of the apoptosis inhibition action exerted by D-Asp, an increase in apoptosis in the seminiferous tubules in the absence of D-Asp in Ddo knock-in mice was demonstrated, as highlighted by the higher levels of the cytosolic protein Cyt c and by an increase in the percentage of TUNEL-positive cells compared to the wild-type [23].

Therefore, D-Asp seems to inhibit the apoptotic process in the testis. This effect could occur through a reduction in oxidative stress.

## 6. D-Asp Improves the Capacity of Sertoli Cells to Sustain Spermatogenesis 

Sertoli cells are testicular somatic cells that regulate spermatogenesis by performing crucial supportive functions for spermatogenic cells. The number of SCs limits the sperm production capacity [78]. Insufficiency and impaired function of these cells are recognized as contributing factors to male infertility [79,80]. The potential role of D-Asp in SC has been overlooked until recently. A recent in vitro study investigated the effects of D-Asp on TM4 SCs, a non-tumorigenic cell line derived from the testes of 11- to 13-day-old mice [30]. TM4 cells share several characteristics with SCs, including the expression of follicle-stimulating hormone (FSH), androgen receptor (AR), and progesterone receptor [81]. The addition of D-Asp into the culture medium resulted in the increased proliferation and activity of TM4 cells, demonstrated by the activation of the ERK/AKT/PCNA pathway and enhanced levels of AR [30] (Figure 3). Numerous studies have emphasized the involvement of the ERK1/2 pathway in the regulation of SC proliferation, differentiation, and apoptosis induced by sex hormones [36]. Moreover, extensive literature suggests that ERK1/2 also regulates other critical functions of SCs, such as paracrine activity, formation of the blood–testis barrier, and the clearance of apoptotic bodies [36].

D-Asp positively influences TM4 cell function, not only by modulating the mitochondrial compartment but also by reducing ER stress [30]. Specifically, D-Asp induces enhanced mitochondrial function, as indicated by the increased mitochondrial membrane potential (MMP) and elevated expression levels of several OXPHOS complexes (CI, CII, CIV), indicative of heightened oxidative phosphorylation levels (Figure 3). Additionally, D-Asp treatment increased the expression of PGC-1α, NRF-1, and TFAM proteins, which are markers of mitochondrial biogenesis, as well as TOMM20, a receptor on the mitochondrial outer membrane that signifies increased mitochondrial mass [30] (Figure 3). Furthermore, D-Asp promoted mitochondrial dynamics, which is evident from the increased expression of the fusion markers MFN1 and MFN2, alongside the fission marker DRP1 (Figure 3). 

Finally, D-Asp stabilized the MAM association in TM4 cells, as suggested by the increased MNF2 expression [30], thus contributing to cellular homeostasis [82]. The MAM also plays a key role in calcium transport from the ER to the mitochondria. D-Asp treatment increased the GRP75 and VDAC levels in TM4 cells, which could favor calcium transfer [83] (Figure 3). Furthermore, a function of the amino acid in the inhibition of ER stress signals responsible, in turn, for the stabilization of the MAM structure and the decrease in apoptosis [84] is suggested by a decrease in GRP78 in TM4 cells treated with D-Asp (Figure 3). Finally, MAMs that participate in lipid transportation between the ER and the mitochondria are involved in lipid metabolism [30]. The protein levels of FACL4 and SOAT1, enzymes involved in lipid biosynthesis, were significantly greater in the TM4 cells treated with D-Asp (Figure 3).

The above data demonstrate a direct effect of D-Asp on SC activity and suggest that D-Asp could improve the capacity of Sertoli cells to sustain spermatogenesis via the ERK1/2/AKT signaling pathways.

## 7. Conclusions 

Decreased testosterone levels are one of the main causes of male infertility and often cannot be resolved with exogenous testosterone, which usually causes the inhibition of LH secretion by the pituitary gland through negative feedback. Thus, one resolution in such cases would be to increase endogenous testosterone. In this regard, numerous studies have indicated that D-Asp, an amino acid endogenously present in the testicular parenchyma and pituitary gland, promotes testosterone biosynthesis. This review reports the cellular/molecular mechanisms elicited by D-Asp in testicular cells, revealing the role of this amino acid as a regulator of testis activity. D-Asp promotes spermatogenesis, not only by enhancing testosterone biosynthesis, but also by triggering autocrine and/or paracrine signaling in testicular cells, which are mediated by the GluR/ERK pathway. Specifically, in Leydig cells D-Asp is involved in several steps of steroidogenesis, all of which follow one another to regulate the synthesis and/or release of testosterone via the cAMP/ERK1/2 signaling pathway. D-Asp also contributes to the success of the spermatogenetic process by promoting the ERK/AKT/PCNA pathway via GluRs (NMDAR and AMPAR) in the testicular germ cells (spermatogonia and spermatocytes) and somatic cells (Leydig and Sertoli cells). In the spermatogonia and spermatocytes, ERK activates the expression of the proteins involved in mitotic and meiotic processes (AuroraB, pH3, SYCP3), respectively. In addition, D-Asp plays a critical role in the testis mitochondrial functionality and MAM stability as well as in cytoskeleton remodeling, a process occurring during cell division. A direct effect of D-Asp on Sertoli cell activity suggests that D-Asp could improve the capacity of Sertoli cells to sustain spermatogenesis via the ERK1/2/AKT signaling pathways. Finally, D-Asp regulates the testicular oxidative state and inhibits the apoptotic process via the AMPAR/AKT pathway.

This review aims to identify the molecules capable of promoting spermatogenesis through the activation of endogenous testosterone biosynthesis. In this context, D-amino acids could be good candidates. Recent studies have shown that D-Ser, which is endogenously present in the testis, also activates germ cell proliferation/maturation [31,32,33]. Undoubtedly, the theoretical basis provided here will be of immense help to researchers working in the field of D-amino acids.
